# Peak expiratory flow rate and chronic respiratory symptoms among restaurant workers: a cross-sectional study from Thailand

**DOI:** 10.12688/f1000research.20059.2

**Published:** 2019-12-02

**Authors:** Chudchawal Juntarawijit

**Affiliations:** 1 Department of Natural Resources and Environment, Faculty of Agriculture, Natural Resources and Environment, Naresuan University, Phitsanulok, 65000, Thailand

**Keywords:** Indoor air pollution, cooking smoke, restaurant workers, peak expiratory flow rate, chronic respiratory symptoms, lung function

## Abstract

**Background**: Cooking fumes are a major source of indoor air pollution affecting millions of people worldwide. To date, there has been no epidemiological study to show the variation in health effects resulting from work at different kinds of restaurants in Thailand. This study determines lung function and chronic respiratory symptoms of workers in four types of eateries commonly found in Thailand.

**Methods**: This is a cross-sectional study of 321 people working in four common types of restaurants in Thailand: **‘tamsang’** restaurants (from the Thai word ร้านอาหารตามสั่ง, a restaurant that makes a variety of foods to order) (170 people), papaya salad restaurants (51 people), noodle restaurants (50 people), and barbecue stalls (50 people).  The restaurant workers’ demographic data as well as information on their working conditions was collected using a questionnaire administered in a face to face interview. Each worker’s peak expiratory flow rate was measured using a portable peak flow meter.

**Results**: This study found that compared to the other three types of restaurants, working in a ‘tamsang’ restaurant has more adverse health effects. Participants from ‘tamsang’ restaurant were at greater  of poor lung function (OR = 2.59, 95% CI 1.33–5.06) and  moderate dyspnea symptoms (OR = 3.79, 95% CI 1.63–8.79) compared to participants  from papaya salad restaurant. The study also found that each of the following were associated with poor lung function and/or chronic respiratory symptoms: cooking with palm oil, having irritated teary eyes while cooking, cooking without a ventilation hood, long past experience working at restaurants, and working in a small cooking area (1–6 m ^2^).

**Conclusions**: Work in different kinds of restaurants with variations in cooking methods and work conditions produces diverse effects on airway and lung function. Regulatory organizations should pay careful attention to protecting the health of restaurant workers, especially those working in ‘tamsang’ restaurants.

## Introduction

Recently cooking fumes have received increased public attention as an indoor and outdoor source of air pollution. The World Health Organization estimated that in 2018 inefficient cooking using solid fuels (biomass, kerosene and coal) caused premature death of about 4 million people worldwide ^1^. Besides smoke from burning fuel, high temperature treatment of food will generate fumes from the degradation of sugars and fats, as well as from pyrolysis of proteins and amino acids. Previous studies have clearly established that cooking fumes commonly contain fine particulate matter and many other toxic compounds, including volatile organic compounds (VOCs), polycyclic aromatic hydrocarbons (PAHs), aldehydes, alkanoic acids, nitrogen dioxide (NO _2_), and carbon monoxide (CO) ^2– 4^. The exact composition and concentration of cooking fumes depend on several factors, including cooking temperature, cooking methods, and cooking oil/fuel type ^5^. Other research has reported that the cooking method releasing the most particles, especially in the ultrafine size range, is deep frying, followed by regular frying, stir frying, boiling, and steaming ^6, 7^. Concentrations of volatile organic compounds can vary from 257.5 to 3,494.0 µg/m ^3^, depending on cooking style ^3^. A study of two Chinese cooking styles found concentrations of fine particles, mostly fatty acids, to be 1,406.3 ± 293.4 µg/m ^3^ for Hunan cooking and 672.0 ± 295.8 µg/m ^3^ for Cantonese cooking ^4^. It has been estimated that cooking with natural gas could add 21–30% to weekly average indoor concentrations of CO and 25–39% to those of NO _2,_ depending on ventilation and season of the year ^8^.

People working in a kitchen, whether at home or in a restaurant setting, are at risk of exposure to cooking fumes and related health consequences. A study of people using natural gas to cook at home without a ventilation hood predicted that they would be exposed to NO _2, _CO, and formaldehyde at 62%, 9%, and 53% above established safety limits, respectively ^8^. Lai *et al*. ^9^ reported a close correlation between levels of toxic chemicals in restaurant air and levels of those same chemicals in the urine of chefs. Exposure to cooking fumes can result in various kinds of respiratory problems. A recent study from Taiwan reported an increased risk of lung cancer among chefs cooking Chinese food ^10^. In places that tend to have modern kitchens, such in Norway, an elevated level of respiratory complaints and chronic bronchitis was also found among professional chefs, especially those who performed a lot of frying ^11^. Studies from various parts of the world have reported an elevated prevalence of both acute and chronic respiratory symptoms and diseases among people involved in cooking ^12– 18^.

Lung function is another effect that has been associated with cooking fumes. A large study of elementary school children found reductions of peak expiratory flow up to 3.4% among children in families cooking with gas ^19^. A study of Chinese restaurants in Hong Kong reported that workers cooking with gas had poorer lung function than those cooking with electricity ^13^. A similar result was also observed in a study from India ^2^. In Nigeria, workers exposed to wood smoke and oil fumes from a local style of grilled meat called “mai suya” have lower forced expiratory volume in one second (FEV1) and lower forced vital capacity (FVC) compared to an unexposed control group ^18^.

In Thailand, four common types of eateries are: **‘tamsang’** restaurants (a kind of Thai restaurant that makes a variety of foods to order, from the Thai word ร้านอาหารตามสั่ง), noodle restaurants, papaya salad restaurants, and roadside barbecue stalls ^20^ The kinds of food available at these four sorts of eateries are not the same. Of the four, ‘tamsang’ restaurants sell the widest variety of food, mainly popular Thai dishes, including red curry, basil stir fry, fried rice and numerous other fried dishes. Most of the food from ‘tamsang’ restaurants is cooked at high temperatures, creating a lot of oily steam and pungent vapors from frying chili peppers and other spicy ingredients. In noodle restaurants, much of the food is boiled but some noodle restaurants also make fried dishes, like phat Thai and other fried noodle dishes. At roadside barbecue stalls, various kinds of meat (beef, pork, chicken, and seafood) are grilled, usually over charcoal, producing abundant smoky vapors. In papaya salad restaurants, the salad is often served with some side dishes, such as sour soup, spicy meat salad, and grilled chicken. Therefore, papaya salad restaurants do employ a variety of cooking methods, including boiling, frying, grilling, but there is relatively less frying than at a ‘tamsang’ restaurant and less grilling than at a barbecue stall ^20^.

To date, there has been no epidemiological study to show the variation in health effects resulting from work at different kinds of restaurants in Thailand. A previous study did find a higher occurrence of respiratory symptoms among restaurant workers in general, but due to small sample size, the study was not able to reveal a statistically significant difference in health effects between different types of restaurants ^17^. The current study investigates peak expiratory flow rate (PEFR) and chronic respiratory symptoms among workers at different types of restaurants in Thailand. The study results are useful for the prevention and control of occupational health problems among restaurant workers. The purpose of this study is to investigate if cooks working in different types of kitchens have different rates of impaired lung function and occurrence of respiratory symptoms.

## Methods

This study is a cross-sectional study.

### Study site

Phitsanulok is a medium size province in northern Thailand, about 400 km from Bangkok, with a population of 865,368 in 2017, and its largest city is Phitsanulok City. In 2017, Phitsanulok City reported that there were 2,511 restaurants within the city limits (personal communication, December 15, 2017).

### Study participants

In this study, a cluster sampling technique was employed by first divided restaurant types into four groups (‘tamsang’ restaurant, noodle restaurant, papaya salad restaurant, and barbecue stall), and then, individual restaurants were chosen using the consecutive sampling technique, where all the restaurants meeting the criteria were selected until the required sample size was reached. A total of 321 participants (170 from ‘tamsang’ restaurants, 50 from noodle restaurants, 51 from papaya salad restaurants, and 50 from barbecue stalls) were selected, this proportion roughly representing the frequency with which each restaurant type is encountered in the city. At each restaurant, one worker, preferably a chef who was at least 20 years old and willing to participate in the study, was interviewed and measured for PEFR.

The sample size calculation for a proportion or descriptive study was used to calculate the necessary sample size which found to be 322 using OpenEpi (version 3.0). The sample size calculation employed the following assumptions: population size = 2,500 (based on data from Phitsanulok City Hall), proportion of population with outcome = 0.40 (based on the previous study of the author, which found a prevalence of respiratory symptoms to be about 12% to 54% ^17^, confidence interval = 95%, and standard error = 0.05.

### Study questionnaire

A questionnaire administered by interviewers was used to collect data in face-to-face interviews with restaurant workers (provided as extended data in English and Thai ^21^). The interviews took place in a restaurant. The data was collected during the period of January–March 2018, when the weather is dry and mild in Thailand and therefore is unlikely to affect the respiratory health of the participants. The questionnaire was identical to that in a previous study by the current author on respiratory symptoms among restaurant workers ^17^. The questionnaire was not validated but before use, it was tested for question sequencing and understanding. In addition to demographic data, the study also collected information on cooking fuel, cooking oil, kitchen size (approximated by interviewer) and types, use of ventilation hood, and frequency of tears while cooking (TWC), tearing eyes caused by smoke exposure.

The questions asked on chronic respiratory symptoms were developed based on British Medical Research Council (Medical Research Council Questionnaire, MRCQ) ^22^ and American Thoracic Association (ATS-DLD-78) questionnaires ^23^. Information on respiratory symptoms that were collected in this study included: chronic cough, chronic phlegm, wheeze, moderate dyspnea, and severe dyspnea. People with ‘chronic cough’ refers to those who cough with or without phlegm at least 4 to 6 times a day for 4 or more days out of the week. ‘Chronic phlegm’ refers to people who have sputum for at least twice a day for 4 or more days per week. ‘Wheeze’ refers to those who breathe with a whistling sound whether or not they have a cold. ‘Moderate dyspnea’ refers to people with shortness of breath when walking briskly or exercising. ‘Severe dyspnea’ refers to people with shortness of breath even when undertaking ordinary daily activities.

After each questionnaire interview, the peak expiratory flow rate (PEFR) of each participant was measured using a portable peak flow meter from MicroPeak (MPE8200EU), CareFusion Company, United Kingdom. The meter can measure flow rates in the range of 60–900 L/min, with an accuracy of ±5% (10 L/min). The PEFR of each study participant was measured three consecutive times, and the highest reading was selected to be their PEFR record. This figure was then used to compare with a standard PEFR of Thai people, and study participants with a PEFR of less than 80% of the standard are considered to have abnormal lung function ^24^.

The study data was collected by two graduate students from Naresuan University’s Environmental Sciences master degree program.

### Data analysis

Data was analyzed using IBM SPSS (version 19) software. Frequency distribution of the data on demographics, cooking fumes exposure, PEFR, and chronic symptoms was analyzed using descriptive analysis. The associations between restaurant types and PEFR/ respiratory symptoms were analyzed using multiple logistic regression. The analysis was adjusted for gender (male, female), age (continuous), body mass index (BMI) (continuous), tobacco use (current smoker, ex-smoker, never smoked), and cooking at home (almost always, sometimes, rarely). All analyses were two-sided, with a 95% confidence interval, and a p-value of <0.05 was considered to be statistically significant.

### Ethical statement

Written informed consent was obtained from each study participant before the interviewing process. At that time, study participants were informed of the study’s purposes, the data collection procedure, and their right to refuse participation in the study. This study was approved in advance by the Naresuan University Board of Ethics (Certificate of Approval (COA) number: 033/2018).

## Results

Most of the study participants were female cooks with a similar mean age across all four types of restaurants studied. Most of the participants had been working for more than one year. Less than 20% of the participants were current cigarette smokers. Additional information on the demographic data is shown in Table 1 and underlying data ^25^.

**Table 1.** Demographic data.

	‘Tamsang’	Noodle	Papaya Salad	Barbecue stall	P-value **
No of participants (n = 321)	170	50	51	50	
**Gender**					0.004 *
Men	38 (22.4))	19 (38.0)	10 (19.6)	22 (44.0)	
Women	132 (77.6)	31 (62.0)	41 (80.4)	28 (56.0)	
**Age,** years					0.003 *
20–29	9 (5.3)	9 (18.0)	2 (3.9)	6 (12.0)	
30–39	48 (28.2)	11 (22.0)	17 (33.3)	14 (28.0)	
40–49	34 (20.0)	15 (30.0)	17 (33.3)	6 (12.0)	
50–59	56 (32.9)	13 (26.0)	11 (21.6)	15 (30.0)	
60–69	22 (12.9)	2 (4.0)	4 (7.8)	6 (12.0)	
70–79	1 (0.6)	0 (0.0)	0 (0.0)	3 (6.0)	
Mean ± SD	46.52 ± 11.67	42.04 ± 11.42	44.02 ± 9.81	46.34 ± 13.20	0.08
**Marital status**					0.60
Single	26 (15.3)	9 (18.0)	11 (21.6)	9 (18.0)	
Married	122 (71.8)	39 (78.0)	36 (70.6)	36 (72.0)	
Divorced/spouse passed away/separated	22 (12.9)	22 (4.0)	4 (7.8)	5 (10.0)	
**Body mass index (BMI**) kg/m ^2^					0.046 *
<18.5	7 (4.2)	4 (8.2)	1 (2.0)	4 (8.2)	
18.5–22.9	48 (28.6)	17 (34.7)	17 (33.3)	15 (30.6)	
23.0–24.9	41 (24.4)	5 (10.2)	8 (15.7)	3 (6.1)	
25.0–29.9	45 (26.8)	17 (34.7)	13 (25.5)	22 (44.9)	
≥30	27 (16.1)	6 (12.2)	12 (23.5)	5 (10.2)	
Mean ± SD	25.10 ± 4.79	24.68 ± 4.28	26.07 ± 5.21	25.09 ± 4.40	0.49
**Education completed**					
No school	2 (1.2)	0 (0.0)	5 (9.8)	2 (4.0)	0.003 *
Primary school	73 (42.9)	13 (26.0)	17 (33.3)	19 (38.0)	
High school	85 (50.0)	27 (54.0)	22 (43.1)	23 (46.0)	
University/college	10 (5.9)	10 (20.0)	7 (13.7)	6 (12.0)	
**Tobacco use**					0.90
Current smoker	29 (17.1)	7 (14.0)	7 (13.7)	9 (18.0)	
Ex-smoker	11 (6.5)	5 (10.0)	2 (3.9)	3 (6.0)	
Never smoked	130 (76.5)	38 (76.0)	42 (82.4)	38 (76.0)	
**Job description**					0.04 *
Cook	154 (90.6)	50 (100.0)	49 (96.1)	49 (98.0)	
Other (waitperson, chef assistant)	16 (9.4)	0	2 (3.9)	1 (2.0)	
**Years of working**					0.11
<1	4 (2.4)	4 (8.0)	4 (7.8)	6 (12.0)	
1–4	35 (20.6)	16 (32.0)	14 (27.5)	18 (36.0)	
5–9	39 (22.9)	8 (16.0)	9 (17.6)	8 (16.0)	
10–20	57 (33.5)	15 (30.0)	13 (25.5)	12 (24.0)	
21 or more	35 (20.6)	7 (14.0)	11 (21.6)	6 (12.0)	
Cooking at home					<0.001 *
Almost always	101 (59.4)	19 (38.0)	19 (38.0)	26 (51.0)	
Sometimes	54 (31.8)	21 (42.0)	13 (26.0)	19 (37.3)	
Rarely	15 (8.8)	10 (20.0)	18 (36.0)	6 (11.8)	

*Significant difference between types of restaurants with p<0.05 (2-sided)

**Chi-square test for category data, and ANOVA for continuous variable

It was found that restaurant workers had a high variation in lung function performance with the lowest average peak expiratory flow rate (PEFR) of 278 ± 113 among those working in ‘tamsang’ restaurants and the highest PEFR of 356 ± 107 among workers in noodle restaurants ( Table 2). Compared to standard PEFR values of Thai people, 41.2%–64.7% of restaurant workers had a poor PEFR, defined as PEFR (measured)/PEFR (standard) less than 80%, and the prevalence of poor PEFR varied significantly across the types of restaurants (p<0.001). Many of the workers reported having chronic cough, phlegm, wheezing, moderate dyspnea, and severe dyspnea, but only moderate dyspnea showed a variation among types of restaurants.

**Table 2.** Prevalence of poor peak expiratory flow rate (PEFR) and chronic respiratory symptoms of workers in different types of restaurants.

	‘Tamsang’	Noodle	Papaya salad	Barbecue	P-value
N	170	50	51	50	
PEFR, Mean ± SD	278.05 ± 113.80	356.73 ± 107.96	331.75 ± 86.54	337.43 ± 125.54	<0.001 *
PEFR <80%, n (%)	110 (64.7)	22 (44.0)	21 (41.2)	25 (50.0)	0.004 **
**Chronic symptoms**					
Cough	33 (19.4)	12 (24.0)	14 (27.5)	6 (12.0)	0.24
Phlegm	27 (15.9)	4 (8.0)	4 (7.8)	7 (14.0)	0.31
Wheezing	29 (17.1)	4 (8.0)	6 (11.8)	10 (20.0)	0.28
Moderate dyspnea	69 (40.6)	7 (14.0)	8 (15.7)	13 (26.0)	<0.01 *
Severe dyspnea	27 (15.9)	7 (14.0)	13 (25.5)	11 (22.0)	0.32

*Significant difference between types of restaurants, with p <0.05 (2-sided), ANOVA test

**Significant difference between types of restaurants, with p <0.05 (2-sided), Chi-square test

Further analysis using multiple logistic regression showed that those who worked in ‘tamsang’ restaurants are at a significantly higher risk (p <0.05) of having poor PEFR (OR = 2.59, 95% CI 1.33–5.06), and moderate dyspnea (OR = 3.79, 95% CI 1.63–8.79) compared to papaya salad restaurant workers ( Table 3). A higher risk of poor PEFR and/ or chronic respiratory symptoms were also significantly associated (p <0.05) with the use of palm oil for cooking, with having frequent TWC, use of a ventilation hood, and with working in 1–6 m ^2^ kitchen. All analyses were carried out using the following covariates: age, gender, BMI, cigarette smoking, and cooking at home.

**Table 3.** Odd ratio (OR) ^a^ with 95% confidence interval of low peak expiratory flow rate (PEFR)/chronic respiratory symptoms, by predictive factors.

	Low PEFR	Cough	Phlegm	Wheezing	Moderate Dyspnea	Severe Dyspnea
**Restaurant type**						
Tamsang	2.59 (1.33–5.06)	0.64 (0.30–1.37)	2.18 (0.70 –6.85)	1.69 (0.65–4.42)	3.79 (1.63–8.79)	0.45 (0.20– 0.99)
Noodle	0.91 (0.39–2.10)	0.72 (0.28–1.88)	0.84 (0.18–3.92)	0.65 (0.17–2.56)	0.96 (0.31–2.98)	0.49 (0.17–1.42)
Barbecue	1.48 (0.63–3.45)	0.34 (0.11–1.04)	1.69 (0.41–6.94)	1.95 (0.60–6.31)	2.20 (0.76–6.33)	0.59 (0.21–1.65)
Papaya Salad	1.0	1.0	1.0	1.0	1.0	1.0
**Cooking oil** ^b^						
Soybean	0.98 (0.30–3.22)	0.53 (0.13–2.13)	1.35 (0.22–8.50)	0.68 (0.16– 2.86)	0.36 (0.10–1.23)	1.23 (0.24–6.48)
Palm	5.38 (1.4–20.31)	0.65 (0.16–2.68)	2.97 (0.46–19.27)	0.74 (0.17–3.22)	0.99 (0.28–3.53)	0.73 (0.12–4.41)
Lard	1.0	1.0	1.0	1.0	1.0	1.0
**Tears while cooking (TWC)** ^b^						
Often	3.07 (1.09– 8.63)	10.28 (2.61–40.46)	3.21 (0.68–15.09)	4.08 (1.10 – 15.22)	3.42 (1.20– 9.70)	1.59 (0.46–5.48)
Sometimes	2.80 (1.29–6.06)	1.60 (0.50–5.14)	1.51 (0.44–5.18)	1.46 (0.49–4.35)	1.74 (0.78–3.70)	0.42 (0.14–1.26)
Rarely	1.0	1.0	1.0	1.0	1.0	1.0
**Job description**						
Cook	1.23 (0.45–3.34)	1.12 (0.30–4.13)	NA	0.74 (0.20–2.80)	0.58 (0.21–1.56)	0.33 (0.11–0.98)
Other	1.0	1.0	1.0	1.0	1.0	1.0
**Use of ventilation hood**						
Not used	0.90 (0.52–1.55)	2.25 (1.03–4.94)	1.02 (0.45–2.31)	1.00 (0.48–2.07)	0.86 (0.48–1.52)	2.58 (1.14–5.86)
Used	1.0	1.0	1.0	1.0	1.0	1.0
**Kitchen location** ^b^						
Enclosed	1.32 (0.66–2.65)	1.41 (0.59–3.41)	0.52 (0.17–1.56)	2.42 (0.99–5.90)	1.30 (0.65–2.58)	0.47 (0.17–1.32)
Outdoor	1.0	1.0	1.0	1.0	1.0	1.0
**Ventilation and kitchen** **location** ^b^						
Enclosed and use ventilation hood	0.72 (0.18–2.96)	NA	NA	2.42 (0.43–13.60)	0.41 (0.07–2.23)	NA
Outdoor and not use ventilation hood	1.0	1.0	1.0	1.0	1.0	1.0
**Kitchen area** ^b^, m ^2^						
1 – 6	2.22 (1.13– 4.36)	0.71 (0.30–1.66)	1.20 (0.46–3.18)	1.63 (0.69–3.92)	0.74 (0.38–1.43)	0.62 (0.25–1.55)
≥7	1.0	1.0	1.0	1.0	1.0	1.0
**Years of working**						
11 or more	0.98 (0.48–1.99)	1.21 (0.45–3.22)	1.12 (0.31–4.10)	4.74 (1.22–18.46)	0.61 (0.27–1.39)	0.62 (0.26–1.47)
5–10	0.85 (0.44–1.65)	1.37 (0.57–3.26)	2.33 (0.77–7.11)	2.75 (0.82–9.18)	0.55 (0.24–1.25)	1.01 (0.46–2.26)
1–4	1.0	1.0	1.0	1.0	1.0	1.0

^a^Adjusted for: Gender (male vs. female), age (continuous), body masss index (BMI) (continuous), tobacco use (current smoker, ex-smoker, never smoked), cooking at home (almost always, sometimes, rarely)

^b^Analysis using only data from ‘tamsang’ restaurant workers

NA = Data not available due to small sample size

## Discussion

This study revealed that a large proportion of restaurant workers have poor PEFR, with the highest frequency of 64.7% found in ‘tamsang’ restaurants and the lowest rate of 41.2% in papaya salad restaurants ( Table 2). Compared to workers in papaya salad restaurants, workers in ‘tamsang’ restaurants are more likely to have a low PEFR (OR = 2.59, 95% CI 1.33–5.06) and moderate dyspnea symptoms (OR = 3.79, 95% CI 1.63–8.79) ( Table 3). These results are consistent with previous literature, which has reported that cooking fumes contains pollutants, such as fine particulate matter, acrolein, formaldehyde, and NO _2_, that can cause airway irritation ^2, 26, 27^. Although no relevant data from Thailand appears to exist, it is expected that Thai style cooking might generate more of these pollutants than other literature has reported in some other styles of cooking, because Thai cooking more often involves stir frying at high temperature, which is similar to Chinese cooking, but Thai cooking often uses more spices and other pungent ingredients. These pollutants are found more often in high temperature frying than in boiling or steaming ^3, 28^, which poses a real hazard to workers at ‘tamsang’ restaurants, since, as mentioned earlier, ‘tamsang’ restaurants do a lot of frying. More pollutants can also be expected from barbecue stalls, which usually grill meat with charcoal. However, this type of eatery is usually located outdoors in the open air, therefore exposure can be greatly reduced by the wind. Also somewhat surprising in this study, ‘tamsang’ restaurant workers were found to have a lower risk of severe dyspnea (OR = 0.45, 95% CI 0.20–0.99). This might be the result of the so called ‘healthy worker effect’ (HWE), in which people who have severely illness are excluded from the job ^29^. Similar findings were also observed in the association between working as a cook and prevalence of severe dyspnea (OR = 0.33, 95% CI 0.11–0.98) ( Table 3). This bias is likely limited to the more severe symptoms like severe dyspnea rather than other symptoms with milder health effects.

This study surprisingly found that compared to cooking with lard, cooking with palm oil is associated with an elevated risk of poor PEFR (OR = 5.38, 95% CI 1.43–20.31) but soybean oil was not ( Table 3). This finding seems to contradict information that palm oil and lard contain mostly saturated and monounsaturated fats, and that both are similarly resistant to oxidation and have a high boiling point ^28, 30^. Compared with soybean oil which has a lower boiling point, lard and palm oil would be expected to produce fewer harmful pollutants when cooked. However, a study by Lee *et al.* ^31^ also found a negative effect from palm olein in the form of higher levels of aldehyde when frying chicken with palm olein compared to other cooking oils, despite similar amounts of fatty acids. At present, data on health effects of palm oil is limited. Further research is therefore needed to investigate this issue.

The current study also found that low PEFR is correlated with tears while cooking (TWC), which refers to tearing eyes caused by smoke exposure, in a dose-response fashion, with an OR of 3.07 (95% CI 1.09–8.63) for those who often had TWC and an OR of 2.80 (95% CI 1.29–6.06) for those who only occasionally had TWC ( Table 3). TWC was also significantly correlated with many chronic symptoms, including cough, wheezing, and moderate dyspnea. This result confirms that TWC is a good marker for cooking fumes. In a previous study of the current author, TWC was also found to be correlated with respiratory symptoms ^17^.

This study also showed that not using a ventilation hood is significantly associated with an increased risk of chronic cough (OR = 2.25, 95% CI 1.03–4.94) and severe dyspnea (OR = 2.58, 95% CI 1.14–5.86) ( Table 3). This finding was consistent with general thinking and previous research that has found that ventilation hoods, even when operating inefficiently, can significantly reduce cooking fumes exposure ^8, 32^. On the other hand, regarding kitchen location, there were no significant associations found. A study in Ghana similarly found that concentrations of PM2.5 and black carbon did not significantly differ across outdoor, enclosed, and semi-enclosed kitchens ^5^.

Inside ‘tamsang’ restaurants, people working in a smaller kitchen area of 1–6 m ^2^ have an increased risk of abnormal PEFR (OR = 2.22; 95% CI 1.13–4.36), compared to those working in a larger kitchen area of ≥7 m ^2^ ( Table 3). One possible explanation is that restaurants with a larger kitchen area tend to have and use ventilation hoods more often than restaurants with a smaller kitchen area. Another possible explanation is that because a large kitchen area has more space, there is a greater volume of air to dilute the pollutants. Further analysis revealed that, in fact, large kitchens use ventilations hoods ***less*** frequently than small kitchens (27.5% vs. 44.3%, respectively). Thus, the first idea can be rejected.

### Study limitations

This study employs a cross-sectional design, in which data on both cooking fumes exposure and health outcome were collected at the same time. Thus, a causal association between the two cannot be drawn. Further study using an alternate design would be useful. Study results might also be adversely affected by small sample size and non-probabilistic sampling technique. This study used data of restaurants located in only one municipality area which may not represent all the restaurants in the whole country. However, all communities in each province of Thailand have similar types of restaurants and cooking style. Although the study data was collected locally and external validity cannot be presumed, the study results would likely be similar in other parts of Thailand, because they present the adverse health effects of a very common Thai cooking style that is widespread throughout the country.

Although the questionnaire was developed with reliable questions from respected medical associations, there is a chance of information bias regarding respiratory symptoms, which were reported by study participants rather than with confirmation from medical doctors.

Apart from cooking fumes, restaurant workers might also be exposed to roadside air pollution, and this could skew results. However, based on data from the Pollution Control Department of Thailand, all six criteria pollutants (PM10, Pb, CO, NO _x_, SO _2_, O _3_) in the Phitsanulok area were within safe air standards.

## Conclusion

Working in a Thai restaurant increases risk of abnormal lung function and chronic respiratory symptoms. Compared to working in a papaya salad restaurant, working in a ‘tamsang’ restaurant is associated with a lower PEFR, and higher rates of moderate dyspnea. Use of palm oil, having frequent tears while cooking, not using a ventilation hood, and working in a small kitchen area are significant predictors of poor lung function and/ or chronic respiratory symptoms. These findings are relevant for anyone concerned with the health and welfare of restaurant workers. Relevant regulatory organizations need to address these dangers in order to protect the health of restaurant workers, particularly those in ‘tamsang’ restaurants.

## Data availability

### Underlying data

Figshare: PEFR and chronic respiratory symptoms. https://doi.org/10.6084/m9.figshare.8980592.v2 ^25^

This project contains the following underlying data:

- PEFR_Chronic_symptoms.sav (Collected demographic and respiratory data)
- Data Dictionary.docx (Word document containing dictionary for study dataset)

### Extended data

Figshare: Questionnaire-PEFR-chronic respiratory symptoms. https://doi.org/10.6084/m9.figshare.9114503.v1 ^21^

This project contains the following extended data:

- Questionnaire-English.docx (Study questionnaire, English)
- Questionnaire-Thai.docx (Study questionnaire, Thai)
