# Supplementary material for: Peak expiratory flow rate and chronic respiratory symptoms among restaurant workers: a cross-sectional study from Thailand
Source: F1000Res. 2019 Dec 2;8:1429. Originally published 2019 Aug 14. [Version 2] doi: 10.12688/f1000research.20059.2 (PMC6904982; doi:10.12688/f1000research.20059.2)
Supplement: Peer review history

# Peer review: Reviewer response for version 2

**DOI:** 10.5256/f1000research.23661.r57353

**Published:** 2020-01-14

**Authors:** Jan G.C. van Amsterdam

**Affiliations:** 1 Department of Psychiatry, Academic Medical Center, University of Amsterdam, Amsterdam, The Netherlands

I confirm that I have read this submission and believe that I have an appropriate level of expertise to confirm that it is of an acceptable scientific standard.

# Peer review: Reviewer response for version 2

**DOI:** 10.5256/f1000research.23661.r57354

**Published:** 2019-12-18

**Authors:** Bjørn Hilt

**Affiliations:** 1 Department of Occupational Medicine, St. Olavs Hospital, Trondheim, Norway

The manuscript now shows considerable improvements and is, in my view, almost ready for indexing.

 Just very few minor points:

 Why is “tamsang” written in bold both in the abstract and the intro?

 The table text of Table 1 could be more self-explanatory than only “Demographic data”.

 In table 1 I am also still a bit unsure as to the meaning of the p-values. For such background data I would not do any significance testing at all, but also understand if the author wants to include it. But, for most variables like gender, age and so on, there is a p-value given as an overall assessment, while for education the p-value is given for the category “no school”.

 For table 2 it is still not clear to me for what differences the p-value is given. In the text it says that it is for the differences between Tamsang and Papaya Salad, and that should also be made clear in the footnotes in the table.

 I still think that the first sentence of the conclusion should be omitted since that is not what the results show if Thai restaurant means all four types of restaurants studied.

 After having reviewed the first version of the manuscript, I felt left with a question of what is so special with the tamsang restaurants, and am no pleased to find the explanation in the second last para of the introduction.

I confirm that I have read this submission and believe that I have an appropriate level of expertise to confirm that it is of an acceptable scientific standard.

# Peer review: Reviewer response for version 2

**DOI:** 10.5256/f1000research.23661.r57355

**Published:** 2019-12-10

**Authors:** Satoshi Nakai

**Affiliations:** 1 Graduate School of Environment and Information Sciences, Yokohama National University, Yokohama, Japan

No comments

I confirm that I have read this submission and believe that I have an appropriate level of expertise to confirm that it is of an acceptable scientific standard.

# Peer review: Reviewer response for version 1

**DOI:** 10.5256/f1000research.22024.r54980

**Published:** 2019-11-12

**Authors:** Jan G.C. van Amsterdam

**Affiliations:** 1 Department of Psychiatry, Academic Medical Center, University of Amsterdam, Amsterdam, The Netherlands

The study of Juntarawijit describes adverse health effects among employees working in four common types of restaurants in Thailand. Main results are that employees of ‘tamsang’ restaurants are more affected by cooking smoke than those working in papaya salad restaurants.

 This is a nice study which suffers from some methodological errors.

 Main points

 The selection method was poorly described. Did all invited participants agree to participate? Part of the participants was no cook. What was their function? Were they also exposed to cooking smoke? What proportion of the participants were allergic? E.g. mites or pollen? Why were for example waiters (not exposed to cooking smoke not used as comparison group? The group ‘Other job’ (Table 1) should be deleted from data analysis, because they are ill characterized and group size is too small.

 Minor points

 Abstract: 

 Please describe that much less adverse effects were seen in the participants in the other three groups. 3rd line: replace ‘health effects’ by ‘adverse health effects’.

 Introduction:

 Please distinguish the effects of cooking smoke vs. methods of heating (coal, gas).

 Give in paragraph 3 some indication of effect sizes observed.

 Methods:

 Study participants, Line 5-6: please specify ‘each restaurant worker’ and use singular in this sentence. Why was only one worker per restaurant included? Any data of homogeneity in effects per restaurant?

 Data from the Pollution Control Department showed that pollutants in the Phitsanulok area were within safe air standards. However, did they vary during the study period? To which extent was traffic related air pollution involved in the outcome? Was any restaurant situated near a busy road (probably it was!)?

 Results:

 Define TWC when first time used. Was TWC related to allergy? Was TWC due to irritating smoke or rather an allergic reaction?

 Please present the results of the combination: ‘Use of ventilation’ and ‘enclosed location’. There may be an adding effect as both affect the indoor air quality.

 Discussion: 

 Probably palm oil and lard give a different heating temperature (frying temperature without burning; ‘boiling point’). Suggest to include this difference to explain the differences found in adverse effects. Similar for soy bean oil.

I confirm that I have read this submission and believe that I have an appropriate level of expertise to confirm that it is of an acceptable scientific standard, however I have significant reservations, as outlined above.

# Response

**Published:** 2019-11-24

**Authors:** Chudchawal Juntarawijit

**Affiliations:** Naresuan University, Thailand

Comment:

Main points: The selection method was poorly described. Did all invited participants agree to participate? Part of the participants was no cook. What was their function? Were they also exposed to cooking smoke? What proportion of the participants were allergic? E.g. mites or pollen? Why were for example waiters (not exposed to cooking smoke not used as comparison group? The group ‘Other job’ (Table 1) should be deleted from data analysis, because they are ill characterized and group size is too small.

Response:

Participant selection method was completely revised.

Yes, all invited participants agree to participate.

Nearly all of the participants are chefs and the rest are waitperson or chef assistant. 

Sorry, we have no data on allergies among participants.

The research data showed that all workers in a restaurant are exposed to cooking smoke.

The number of waitperson is not also enough to be a comparison group.

At first, we plan to collect data from everyone working in a restaurant but the idea reject by the restaurants’ owner. After a negotiation, they allow us to collect data from one worker.

Yes, data on ‘other job’ is too small. However, it is an actual field data and we prefer to present it.

Comment:

Minor points:

Abstract: 

Please describe that much less adverse effects were seen in the participants in the other three groups. 3rd line: replace ‘health effects’ by ‘adverse health effects’.

Response: Abstract was amended as suggestion and the term was revised.

Comment:

Introduction:

Please distinguish the effects of cooking smoke vs. methods of heating (coal, gas).

Give in paragraph 3 some indication of effect sizes observed.

Response:

To avoid confusion, the term “cooking fumes” has been used instead of ‘cooking smoke’ and ‘cooking oil fume’. Cooking fumes are usually from both burning fuel (heating by using coal, gas), and cooking oil fume (COFs), which caused by overheating cooking oil. However, fumes from these two sources cannot be distinguished in field study.

Comment:

Methods:

Study participants, Line 5-6: please specify ‘each restaurant worker’ and use singular in this sentence. Why was only one worker per restaurant included? Any data of homogeneity in effects per restaurant?

Data from the Pollution Control Department showed that pollutants in the Phitsanulok area were within safe air standards. However, did they vary during the study period? To which extent was traffic related air pollution involved in the outcome? Was any restaurant situated near a busy road (probably it was!)?

Response:

The mistakes were corrected and the whole section has been revised.

At the beginning, we planned to collect data from everyone working in a restaurant but the idea was rejected by restaurants’ owners. After a negotiation, they allowed us to collect data from only one worker.

We believe that ambient air and traffic pollution will not much affect the health outcomes even though most of the restaurants situated in a main road. Besides, the fact that level of pollution is still within standard limit, the main purpose of this study was to compare health of workers working in between types of restaurants, which located in the same area.

Comment:

Results:

Define TWC when first time used. Was TWC related to allergy? Was TWC due to irritating smoke or rather an allergic reaction?

Please present the results of the combination: ‘Use of ventilation’ and ‘enclosed location’. There may be an adding effect as both affect the indoor air quality.

Response:

The term TWC has been defined as suggested.

In this study, TWC refers to an acute reaction caused by smoke exposure rather than allergic reaction.

Results of the combination effects of ‘use of ventilation’ and ‘enclosed location’ has been analyzed as suggested. However, it was not found to be significantly associated with PEFR and chronic symptoms. The data was added to Table 3.

Comment:

Discussion: 

Probably palm oil and lard give a different heating temperature (frying temperature without burning; ‘boiling point’). Suggest to include this difference to explain the differences found in adverse effects. Similar for soy bean oil.

Response:

Actually, palm oil and lard did not have a different boiling point. Both have high boiling point as compare to soybean oil. Therefore, it was expected to see similar low health impacts from cooking with palm oil and lard, but more from soybean.

However, issue is interesting, and the discussion has been amended to clarify these points.

# Peer review: Reviewer response for version 1

**DOI:** 10.5256/f1000research.22024.r54898

**Published:** 2019-11-11

**Authors:** Bjørn Hilt

**Affiliations:** 1 Department of Occupational Medicine, St. Olavs Hospital, Trondheim, Norway

With certain limitations, this is an interesting and well-written manuscript focusing on the health of cooks in different types of kitchen in Thailand. The main limitation is of course the cross-sectional design, the description of the selection of participants, and to some extent also the local approach (see below).

 The introduction is very good and covers most of the adequate knowledge in the field. If the author want to seem entirely up to date, they could also have included this very recent reference: Sindre Rabben Svedahl, Bjørn Hilt, Kristin Svendsen. Work environment factors and respiratory complaints in Norwegian Cooks. Int Arch Occup Environ Health. 2019 Sep 17. doi: 10.1007/s00420-019-01473-w. [Epub ahead of print].

 As is common, the author use operational criteria in the description of the objectives of the study. The research question is not whether cooks differ in PEFR, but if they have impaired lung function related to work-exposures. A better and a more conceptual way to state the objectives would in my view be: “To investigate if cooks working in different types of kitchen have impaired lung function and an increased occurrence of respiratory symptoms”. Then to use PEFR as a measure for lung function is legitimate and purely operational.

 The description of the selection of study participants is not absolutely crystal-clear (last para on p3 and first on p4). Somehow, it does not quite make sense. In the very first sentence there seems to be a misprint in “… by first diveded…” which in my eyes should be “…by first dividing…”. Please forgive my ignorance, but what is a “consecutive sampling technique”? Then the reader (at least this one) gets confused about who was selected from which restaurants. Was it one worker from each restaurant fulfilling certain criteria employees was interviewed?  And, it should preferably be a chef. This does not quite make sense to me. Does it mean that they visited 321 restaurants and interviewed one person in each? This needs to be made clearer so that even this reader can understand it easily.

 Another thing with the selection of subjects is that it might have led to a selection of respiratory healthy cooks free from asthma, COPD and emphysema which again has strengthened the so called “healthy worker effect” that the author come back to in the discussion.

 Some other (mostly minor) points as they occur:

 Introduction last para: Was it for statistical reasons that the previous study failed to demonstrate a significant difference? I would rather say that it was due to small numbers that the study failed to unveil a statistically significant difference.

 P4, 2 ^nd^ para: When we do power assessments we do not calculate the “optimal” sample size, but the “necessary” sample size. What does the last sentence of the para mean? Please clarify.

 In the introduction the author use both “cooking oil fumes (COF)” and “cooking smoke”. Is there a difference between them? For my part I am used to the term “cooking fume”.

 Were the PEFR measurements done at random times of the day? I mean, if all measurements from one type of restaurants were done in the morning and the measurement from another in the evening, it could possibly have biased the results due to circadian differences in lung function.

 Do the author have a literature reference for the expected lung function values for the Thai people? Instead of a dichotomized (>< 80 %) variable for PEFR they could also have given us the actual percentages. In table 2 they give the actual mean values without any adjustments. For those figures with three main figures, I think it is unnecessary to put it with two decimals.

 For table 1 and 2 the reader needs to know the meaning of the column with p-values. For what comparision?

 Table text for table 2 needs to be more comprehensive and explanatory.

 Since table 3 is divided in two, it needs to be stated that it will be continued at the bottom of the first part. For table 3 it is not necessary to explain in a footnote for the reader that a 95% confidence interval that not includes the value 1 is statistically significant on a 5% level.

 I see tears while cooking more at another effect that a real predictor/determinant of the other effects. In the same time it is of course correct to say that if the exposure is so strong that you have the symptom TWC, you can also have other, and worse effects.

 A better reference and explanation of the tables should be given in the text.

 In the first para of the discussion the so called healthy worker effect is noting but selection bias and no phenomenon on its own. Selection bias may well have been introduced since the participants apparently should be free from respiratory diseases.

 The paras on study limitations are insightful and well written. The fact that the study is quite local and that the external validity (generalizability) is limited could also have been included. Even so, it is worthwhile to report on deleterious health effects from professional and domestic cooking from different parts of the world with different cooking methods.

 For the conclusion I believe that the term Thai restaurants is a term for all the four types of restaurants included, while it was in “tamsang” restaurants that the effects were found. Perhaps the first sentence of the conclusion could be omitted and that they only start with “This study shows that employees in “tamsang” restaurants in Thailand have…..”

 The consequences of the results are not worth the attention for “regulatory organizations” only, but for everyone concerned with the health and welfare of restaurant workers.

I confirm that I have read this submission and believe that I have an appropriate level of expertise to confirm that it is of an acceptable scientific standard, however I have significant reservations, as outlined above.

# Response

**Published:** 2019-11-24

**Authors:** Chudchawal Juntarawijit

**Affiliations:** Naresuan University, Thailand

Comment: The introduction is very good and covers most of the adequate knowledge in the field. If the author want to seem entirely up to date, they could also have included this very recent reference: Sindre Rabben Svedahl, Bjørn Hilt, Kristin Svendsen. Work environment factors and respiratory complaints in Norwegian Cooks. Int Arch Occup Environ Health. 2019 Sep 17. doi: 10.1007/s00420-019-01473-w. [Epub ahead of print].

Response: Thank you. The reference was added to the manuscript.

Comment: As is common, the author use operational criteria in the description of the objectives of the study. The research question is not whether cooks differ in PEFR, but if they have impaired lung function related to work-exposures. A better and a more conceptual way to state the objectives would in my view be: “To investigate if cooks working in different types of kitchen have impaired lung function and an increased occurrence of respiratory symptoms”. Then to use PEFR as a measure for lung function is legitimate and purely operational.

Response: Thank you for this helpful insight. The study objective was restated as suggested.

Comment:

The description of the selection of study participants is not absolutely crystal-clear (last para on p3 and first on p4). Somehow, it does not quite make sense. In the very first sentence there seems to be a misprint in “… by first diveded…” which in my eyes should be “…by first dividing…”. Please forgive my ignorance, but what is a “consecutive sampling technique”? Then the reader (at least this one) gets confused about who was selected from which restaurants. Was it one worker from each restaurant fulfilling certain criteria employees was interviewed?  And, it should preferably be a chef. This does not quite make sense to me. Does it mean that they visited 321 restaurants and interviewed one person in each? This needs to be made clearer so that even this reader can understand it easily.

Response:

The description of participant selection was completely revised. 

The term “by first divided” was change to “by first dividing”.

Yes, all 321 restaurants were visited and one person was interviewed at each shop.

Actually, the study wanted to interview every worker in a restaurant, but most restaurants refused to do that way. After negotiation, only one participant, preferring to be a chef, was interviewed.

Most of restaurants in Thailand are small size and they usually have only one chef, the rest is waitperson and chef assistance.

Another thing with the selection of subjects is that it might have led to a selection of respiratory healthy cooks free from asthma, COPD and emphysema which again has strengthened the so called “healthy worker effect” that the author come back to in the discussion.

I agree. From the field experience, we found no workers with those chronic diseases and thus, to be excluded from this study. Therefore, we decide to delete the criteria to avoid confusion.

Comment:

Introduction last para: Was it for statistical reasons that the previous study failed to demonstrate a significant difference? I would rather say that it was due to small numbers that the study failed to unveil a statistically significant difference.

Response: The sentence has been revised as suggested.

Comment: P4, 2 ^nd^ para: When we do power assessments we do not calculate the “optimal” sample size, but the “necessary” sample size. What does the last sentence of the para mean? Please clarify.

Response: Thank you for the reminding. The word “optimal” has been replaced with “necessary”.

Comment:

In the introduction the author use both “cooking oil fumes (COF)” and “cooking smoke”. Is there a difference between them? For my part I am used to the term “cooking fume”.

Response:

You are right. The term “cooking smoke” and cooking oil fume (COFs) has been replaced with “cooking fumes”.

Comment:

Were the PEFR measurements done at random times of the day? I mean, if all measurements from one type of restaurants were done in the morning and the measurement from another in the evening, it could possibly have biased the results due to circadian differences in lung function.

Response:

Yes, the PEFR measurements of each type of restaurants were done at random times of the day, and the bias should be minimal.

Comment:

Do the author have a literature reference for the expected lung function values for the Thai people? Instead of a dichotomized (>< 80 %) variable for PEFR they could also have given us the actual percentages. In table 2 they give the actual mean values without any adjustments. For those figures with three main figures, I think it is unnecessary to put it with two decimals.

Response:

Yes, we do have a reference for expected lung function of Thai people. To measure PEFR percentage, participants have to perform lung function test and the measured PEFR was then compared with PEFR standard which depend on gender, age, and height of the participant.

We agree that more data should be presented. So, we decide to add data on average standard PEFR, and data on distribution of PEFR percent.

The decimals was deleted.

Comment:

For table 1 and 2 the reader needs to know the meaning of the column with p-values. For what comparision?

Response:

More information on p-values was added to make sure that the readers known what the comparisons are.

Table text for table 2 needs to be more comprehensive and explanatory.

The table text was completely revised.

Comment:

Since table 3 is divided in two, it needs to be stated that it will be continued at the bottom of the first part. For table 3 it is not necessary to explain in a footnote for the reader that a 95% confidence interval that not includes the value 1 is statistically significant on a 5% level.

Response: All the suggestions were taken, and the footnote was deleted.

Comment: I see tears while cooking more at another effect that a real predictor/determinant of the other effects. In the same time it is of course correct to say that if the exposure is so strong that you have the symptom TWC, you can also have other, and worse effects.

Response: I agree that TWC can be either effect of its own or a predictive factor. However, in this study, TWC was used as a marker of smoke exposure.

Comment: A better reference and explanation of the tables should be given in the text.

Response: Thank you for the suggestion. The paper was revised in many places, e.g. table 2 text.

Comment:

In the first para of the discussion the so called healthy worker effect is noting but selection bias and no phenomenon on its own. Selection bias may well have been introduced since the participants apparently should be free from respiratory diseases.

Response:

I agree that by using the selection criteria the bias was introduced. However, healthy worker effect is difficult to avoid unless control group with similar health was used as a comparison group. As in this study, health of workers in different types of restaurant was compared to each other, thus HWE will have not much an effect to the study result.

Comment:

The paras on study limitations are insightful and well written. The fact that the study is quite local and that the external validity (generalizability) is limited could also have been included. Even so, it is worthwhile to report on deleterious health effects from professional and domestic cooking from different parts of the world with different cooking methods.

Response: Thank you for your thoughtful and generous notion. The idea was added to the limitation statement.

Comment: For the conclusion I believe that the term Thai restaurants is a term for all the four types of restaurants included, while it was in “tamsang” restaurants that the effects were found. Perhaps the first sentence of the conclusion could be omitted and that they only start with “This study shows that employees in “tamsang” restaurants in Thailand have…..”

Response: It may not true that the effects were found only in “tamsang” restaurant.  The study results showed that 41.2% to 64.7% of the workers have poor PEFR. However, the problem was more pronounce in “tamsang” restaurant.

Comment: The consequences of the results are not worth the attention for “regulatory organizations” only, but for everyone concerned with the health and welfare of restaurant workers.

Response: Thank you for the insightful suggestion. The idea was added to the conclusion.

# Peer review: Reviewer response for version 1

**DOI:** 10.5256/f1000research.22024.r53449

**Published:** 2019-09-16

**Authors:** Satoshi Nakai

**Affiliations:** 1 Graduate School of Environment and Information Sciences, Yokohama National University, Yokohama, Japan

The author discussed the health effects of cooking smoke at restaurants to workers. It has interesting views and it is valuable to index the manuscript. It should be useful to evaluate and control the indoor air environment of cooking places in restaurants, even though the study information may be limited to Thailand.

 However, several things shown in the following comments should be revised:

1. Some sentences are incomplete. Please check the manuscript again.
2. The reviewer considers that papaya salad restaurants are referenced, so the author had better set the salad restaurants at the end of the list. Readers can understand which type of restaurants the reference is.
3. Does the author have some quantitative information about the concentration inside restaurants in Thailand? The author mentioned the other countries’ situation, but no one can image the environment of Thailand. It is very useful for the readers.
  If not, the author must discuss the environment of the restaurant, and the similarity and difference between Thailand and other countries.
4. Are interviewers and examiners of lung function the same persons? Are the interviews and PEFR test conducted at the same time?
5. Second line of “Data analysis”: What are “frequency variables”? Unclear.
6. The author uses “abnormal lung function”, “poor PEFR”, “low PEFR” in this manuscript. The reviewer considers these are the same. Please consider the usage of these terms.
7. The author shows the covariates in the analysis at “Results”. These should be shown in “Methods”.
8. The author writes “cooking at home” in the covariate list, but no data related to cooking at home are shown. Why? The author discussed the effects other than cooking smoke in restaurants. The above information should be important.
9. Why did the author not include “years of working” in this analysis? The reviewer considers it is related to exposure duration and important as an exposure index.
10. The author mentioned the healthy worker effect for severe dyspnea. Why not the other outcomes related to HWE? Please add some comments.

I confirm that I have read this submission and believe that I have an appropriate level of expertise to confirm that it is of an acceptable scientific standard, however I have significant reservations, as outlined above.

# Response

**Published:** 2019-11-24

**Authors:** Chudchawal Juntarawijit

**Affiliations:** Naresuan University, Thailand

1. Some sentences are incomplete. Please check the manuscript again.

The manuscript was rechecked and the errors were corrected.

2. The reviewer considers that papaya salad restaurants are referenced, so the author had better set the salad restaurants at the end of the list. Readers can understand which type of restaurants the reference is.

The list was rearranged and papaya salad restaurants were presented last.

3. Does the author have some quantitative information about the concentration inside restaurants in Thailand? The author mentioned the other countries’ situation, but no one can image the environment of Thailand. It is very useful for the readers.

If not, the author must discuss the environment of the restaurant, and the similarity and difference between Thailand and other countries.

Unfortunately no specific information about concentration of smoke pollutants inside restaurants in Thailand appears to exist. However, discussion about the environment of the restaurants was added to the discussion.

4. Are interviewers and examiners of lung function the same persons? Are the interviews and PEFR test conducted at the same time?

Yes, the interviewers and examiners of lung function are the same persons, and the interviews and PEFR test was conducted at the same time.

This information was added to the method section.

5. Second line of “Data analysis”: What are “frequency variables”? Unclear.

The “Data analysis” was duly revised and the term no longer needed to be used.

6. The author uses “abnormal lung function”, “poor PEFR”, “low PEFR” in this manuscript. The reviewer considers these are the same. Please consider the usage of these terms.

The inconsistency issue was corrected by using only one term, “poor PEFR”.

7. The author shows the covariates in the analysis at “Results”. These should be shown in “Methods”.

Thank you for the reminder. The statement was added to “Methods”

8. The author writes “cooking at home” in the covariate list, but no data related to cooking at home are shown. Why? The author discussed the effects other than cooking smoke in restaurants. The above information should be important.

Data on “cooking at home” was added to Table 1.

9. Why did the author not include “years of working” in this analysis? The reviewer considers it is related to exposure duration and important as an exposure index.

Actually, “years of working” is included in the analysis (Table 3), and it was found to have significant association only to wheezing symptoms.

10. The author mentioned the healthy worker effect for severe dyspnea. Why not the other outcomes related to HWE? Please add some comments.

That is a good point. The reason may be that severe dyspnea is a relatively obvious and serious symptom, comparing with the other symptoms. So, severe dyspnea is more likely to affect their job performance.

This comment was added to the discussion.
